# An increased high-mobility group A2 expression level is associated with malignant phenotype in pancreatic exocrine tissue

**DOI:** 10.1038/sj.bjc.6601391

**Published:** 2003-11-25

**Authors:** N Abe, T Watanabe, Y Suzuki, N Matsumoto, T Masaki, T Mori, M Sugiyama, G Chiappetta, A Fusco, Y Atomi

**Affiliations:** 1 First Department of Surgery, Kyorin University School of Medicine, 6-20-2, Shinkawa, Mitaka, Tokyo 181-8611, Japan; 2 Department of Clinical Pathology, Kyorin University School of Medicine, 6-20-2, Shinkawa, Mitaka, Tokyo 181-8611, Japan; 3 Instituto Nazionale dei Tumori Fondazione Senatore Pascale, via M Semmola, Naples 80131, Italy; 4 Dipartimento di Biologia e Patologia Cellulare e Molecolare, c/o Centro di Endocrinologia ed Oncologia Sperimentale del CNR, Università di Napoli ‘Federico II’, via Pansini, 5, Naples 80131, Italy

**Keywords:** HMGA2, pancreatic cancer, RT–PCR, immunostaining

## Abstract

The altered form of the high-mobility group A2 (HMGA2) gene is somehow related to the generation of human benign and malignant tumours of mesenchymal origin. However, only a few data on the expression of HMGA2 in malignant tumour originating from epithelial tissue are available. In this study, we examined the HMGA2 expression level in pancreatic carcinoma, and investigated whether alterations in the HMGA2 expression level are associated with a malignant phenotype in pancreatic tissue. High-mobility group A2 mRNA and protein expression was determined in eight surgically resected specimens of non-neoplastic tissue (six specimens of normal pancreatic tissue and two of chronic pancreatitis tissue) and 27 pancreatic carcinomas by highly sensitive reverse transcriptase–polymerase chain reaction (RT–PCR) techniques and immunohistochemical staining, respectively. Reverse transcriptase–polymerase chain reaction analysis revealed the expression of the HMGA2 gene in non-neoplastic pancreatic tissue, although its expression level was significantly lower than that in carcinoma. Immunohistochemical analysis indicated that the presence of the HMGA2 gene in non-neoplastic pancreatic tissue observed in RT–PCR reflects its abundant expression in islet cells, together with its focal expression in duct epithelial cells. Intense and multifocal or diffuse HMGA2 immunoreactivity was noted in all the pancreatic carcinoma examined. A strong correlation between HMGA2 overexpression and the diagnosis of carcinoma was statistically verified. Based on these findings, we propose that an increased expression level of the HMGA2 protein is closely associated with the malignant phenotype in the pancreatic exocrine system, and accordingly, HMGA2 could serve as a potential diagnostic molecular marker for distinguishing pancreatic malignant cells from non-neoplastic pancreatic exocrine cells.

The high-mobility group A (HMGA) family of proteins in mammals is composed of four proteins: HMGA1a, HMGA1b, HMGA1c, and HMGA2. The former three proteins are encoded by a single functional gene, that is, HMGA1 (formerly HMGI(Y)), while the last one is a product of a separate gene, that is, HMGA2 (formerly HMGI-C) (Manfioletti *et al*, 1991; Johnson *et al*, 1998). High-mobility group A2 has an approximately 50% amino-acid sequence homology with HMGA1, and features an internal 11 amino-acid deletion that characterises HMGA1 (Manfioletti *et al*, 1991; Tallini and Dal Cin, 1999). High-mobility group A2 proteins bind to the minor groove of AT-rich DNA sequences, thereby inducing a bend within the DNA (Thanos and Maniatis, 1992). They cannot initiate transcription, but they can enhance promotor binding of transcription factors (Thanos and Maniatis, 1992; Grosschedl *et al*, 1994; Mantovani *et al*, 1998).

High-mobility group A2 has been shown to be expressed abundantly during embryogenesis, but its expression is either undetectable or remains at low levels in other normal adult tissues (Manfioletti *et al*, 1991; Zhou *et al*, 1995; Rogalla *et al*, 1996; Rommel *et al*, 1997; Hirning-Folz *et al*, 1998), suggesting that HMGA2 plays an important role (or roles) in cell proliferation and/or differentiation. Consistent with this, it has been demonstrated that HMGA proteins are phosphorylated in a cell-cycle-dependent manner (Reeves *et al*, 1991). Functionally, knocking out the HMGA2 gene in mice leads to the pygmy phenotype with characteristic hypoplasia of mesenchymal tissue, thereby confirming the important role(s) of HMGA2 in mammalian growth and development (Zhou *et al*, 1995).

The altered form of the HMGA2 gene, on the other hand, could somehow be related to the generation of human benign and malignant tumours. Rearrangements of the HMGA2 gene, for example, have been frequently observed in benign tumours of mesenchymal origin (Ashar *et al*, 1995; Schoenmarkers *et al*, 1995). In such cases, the gene rearrangements were the consequence of chromosomal translocation involving regions 12q13–15, where the HMGA2 gene is located. The HMGA2 modifications consist of the loss of the carboxyl-terminal tail and its fusion with ectopic sequences (Ashar *et al*, 1995; Schoenmarkers *et al*, 1995). The truncation of HMGA2, rather than its fusion with other genes, has also been shown to be responsible for cell transformation (Fedele *et al*, 1998). This was confirmed in transgenic mice carrying a truncated HMGA2, which developed a giant phenotype together with a marked expansion of the retroperitoneal and subcutaneous white adipose tissues (Battista *et al*, 1999; Arlotta *et al*, 2000). Interestingly, most of these tumours related to the alteration in HMGA2 are of nonepithelial origin. In contrast, only a few data on the expression of HMGA2 in human malignant tumour originating from epithelial tissue are available (Rogalla *et al*, 1997). The overexpression of HMGA2 mRNA has been shown to be closely associated with high histologic grade in breast cancer (Rogalla *et al*, 1997), suggesting that the expression level of the HMGA2 protein/gene could be a potential clinicopathological marker with prognostic implications for a wide range of cancers. To test this possibility, we examined the HMGA2 expression in pancreatic cancers in the present study, and investigated whether alterations in HMGA2 are associated with the malignant phenotype of tumours in pancreatic tissue. To this end, HMGA2 mRNA expression was first analysed by highly sensitive reverse transcriptase–polymerase chain reaction (RT–PCR) techniques. Immunohistochemical detection of HMGA2 protein using a specific antibody was also attempted. Although relatively simple and easy to perform, immunohistochemistry is a potential method of examining whether the expression of a certain protein is specific to tumour cells, because it allows precise correlation of the protein expression with the phenotype of the cells on individual cell basis (Abe *et al*, 2000). In this sense, immunohistochemistry can provide more useful information than other assays by which proteins and/or mRNAs are extracted from tumours; possibly including a mixture of proteins from normal and irrelevant cells such as acinar cells or islet cells of the pancreas in the analysis (Abe *et al*, 2000). Based on the above considerations, we determined HMGA2 protein expression immunohistochemically on surgically resected specimens, normal pancreatic tissue, chronic pancreatitis tissue, and carcinomas of the pancreas.

## MATERIALS AND METHODS

### Tissue samples

The tissue samples were obtained at the time of surgery at the First Department of Surgery, Kyorin University Hospital, between October 1996 and August 2001. Specimens from 27 pancreatic carcinomas (20 primary carcinomas, four liver metastases, two peritoneal metastases, and one lymph node metastasis) and eight non-neoplastic tissues (six normal pancreatic and two chronic pancreatitis tissues) were obtained. In all, 27 carcinomas were histologically diagnosed as 12 well-differentiated tubular adenocarcinomas, six moderately differentiated tubular adenocarcinomas, seven poorly differentiated tubular adenocarcinomas, and two adenosquamous carcinomas (they were evaluated histologically according to the criteria of the Japan Pancreas Society). Normal pancreatic tissues were obtained from either patients who have undergone pancreatectomy due to pancreatic neoplasms or those with gastric cancer who have undergone pancreatectomy for lymph node dissection. In either case, specimens were obtained from a macroscopically healthy region distinct from the neoplastic lesion. All patients gave their informed consent prior to their inclusion in the study. Among the samples, those from 17 pancreatic carcinomas and six non-neoplastic pancreatic tissues were frozen on dry ice immediately after surgical resection for molecular investigation (RT–PCR), and stored at −80°C until use. All the tissue specimens were fixed for immunohistochemical analysis as soon as possible after surgical resection in 4% paraformaldehyde in phosphate-buffered saline (PBS) at 4°C for 14 h, followed by cryoprotection in a graded concentration series of sucrose in PBS. The specimens were then embedded in the OCT compound, frozen, and stored at −80°C until analysis. All the tissue specimens were histologically examined and pathological diagnoses were confirmed.

### RT–PCR analysis

**Figure 1:**
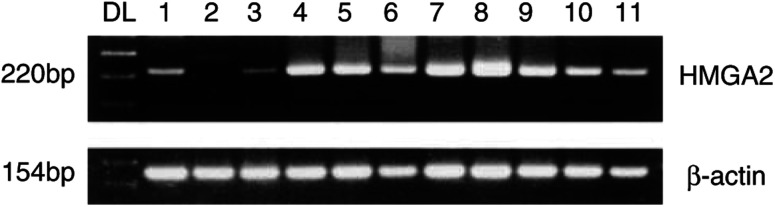
Reverse transcriptase–polymerase chain reaction products of HMGA2 after gel electrophoresis and ethidium bromide staining. Results show specific 220-bp bands. DL, DNA molecular weight marker; lane 1, positive control (hepatoma cell line HEP3B, which is known to express high level of HMGA2); lane 2, normal pancreas; lane 3, chronic pancreatitis; lane 4–11, pancreatic carcinomas.

Reverse transcriptase–polymerase chain reaction for the HMGA2 expression was performed using a heminested PCR technique as described previously (Rogalla *et al*, 1996; Rommel *et al*, 1997). Total RNA was extracted by a modified guanidine thiocyanate method as described previously (Miyatani *et al*, 1986). cDNA was synthesised using the adapter primer (AP) AAG GAT CCG TCG ACA TC (T)17 and Superscript II reverse transcriptase (Gibco BRL, Gaithersburg, MD, USA). For the first and second rounds of the heminested PCR, the same lower primer (Rev) 5′-TCC TCC TGA GCA GGC TTC-3′ (exon 4/5) was used. The forward primer 5′-CTT CAG CCC AGG GAC AAC-3′ (exon 1) and the nested primer 5′-CAT CGC CTC AGA AGA GAG GAC-3′ (exon 1) were used as upper primers. The PCR amplifications were both performed for 30 cycles (1 min at 94°C, 1 min at 53°C, and 2 min at 72°C). As a control reaction for intact RNA and cDNA, PCR amplification of the cDNA of the housekeeping gene *β*-actin was performed for all samples to exclude false-negative PCR results. Only those samples positive for *β*-actin were used for this study. The resulting PCR products were clearly visualised by gel electrophoresis on a 2% agarose-gel stained with ethidium bromide as bands at 220 base pairs (bp) for HMGA2 and at 154 bp for *β*-actin (Figure 1

). The resulting bands were sequenced and their sequences were found to be identical to that of HMGA2.

### Immunohistochemical analysis

Immunohistochemical examinations were performed by the avidin–biotin complex immunoperoxidase technique using an Avidin-Biotinylated Enzyme Complex kit (Vector Laboratories, Inc. CA, USA) as described previously (Abe *et al*, 1999, 2000, 2002). The HMGA2 protein expression was immunohistochemically analysed on surgically resected specimens, together with four pancreatic cancer cell lines (PANC-I, MIA PaCa-2, BxPC-3, and AsPc-1) using HMGA2-specific antibodies, raised in rabbit against the recombinant HMGA2 protein (Berlingieri *et al*, 1995). In brief, frozen sections 5 (5 m thick) were prepared, transferred onto poly-L-lysine-coated slides, air-dried, and then washed in PBS, followed by quenching of endogeneous peroxidase activity with 0.3% hydrogen peroxide in methanol. After further rinsing with PBS, the sections were incubated with normal goat serum for 20 min at room temperature to block nonspecific binding, and then incubated with the primary anti-HMGA2 antibody (1 : 100 dilution) 14 h at 4°C. After another wash in 0.2% Triton X in PBS, the sections were further incubated with biotinylated anti-rabbit IgG for 30 min at room temperature, followed by washes in 0.2% Triton X in PBS. After the addition of streptavidin–biotin-conjugated peroxidase and incubation for 30 min at room temperature, the sections were washed in 0.2% Triton X in PBS, and then the localisation of the HMGA2 protein was visualised by incubating the sections with 3,3′-diaminobenzidine. The slides were counterstained with Mayer's haematoxylin, dehydrated in a graded alcohol series, cleared in xylene, and mounted. Negative control staining was carried out by replacing the primary antibody with normal rabbit serum under the same experimental conditions. The immunostained slides were evaluated microscopically by a single investigator (NA) according to the criteria published previously (Abe *et al*, 1999, 2000) without prior knowledge of the clinical data for each case. The percentage of HMGA2-positive cells was scored by counting approximately 300–1000 tumour cells in three randomly selected fields (Abe *et al*, 1999, 2000, 2002). The immunohistochemical evaluation was considered positive when the HMGA2 nuclear immunoreactivity was detected in more than 20% of the cells according to the criteria published previously (Abe *et al*, 1999, 2000).

## RESULTS

### Expression of HMGA2 mRNA determined by RT–PCR

Among the six non-neoplastic tissue samples, five, including three normal tissues and two chronic pancreatitis tissues, gave rise to detectable HMGA2 bands, while one normal tissue sample showed no detectable HMGA2 band. The signal intensities of the HMGA2 band in chronic pancreatitis tissue samples were almost equivalent to those observed in the normal tissue samples. All the 17 samples of pancreatic carcinomas also showed HMGA2 bands by RT–PCR (Figure 1, Table 1

**Table 1:** HMGA2 expression in pancreatic carcinoma

**Histological type of pancreatic specimens**	**No. of positive specimens/no. of specimens analysed by RT–PCR**	**No. of positive^a^ specimens/no. of specimens analysed by immunohistochemistry**
*Non-neoplastic tissue*		
Normal pancreas	3/4 (75%)	0/6 (0%)
Chronic pancreatitis	2/2 (100%)	0/2 (0%)
		
Duct cell carcinoma	17/17 (100%)	27/27 (100%)

a The immunostained slides were scored as positive for immunohistochemistry when HMGA2 nuclear immunoreactivity was detected in more than 20% of the cells in the exocrine region.

). When the signal intensities of these HMGA2 bands were compared between non-neoplastic and carcinoma samples, the latter showed at least several fold more intense band than the former (Figure 1). Thus, an increased expression level of the HMGA2 mRNA is a distinct feature of pancreatic carcinoma.

### Expression of HMGA2 protein determined by immunohistochemistry

**Figure 2:**
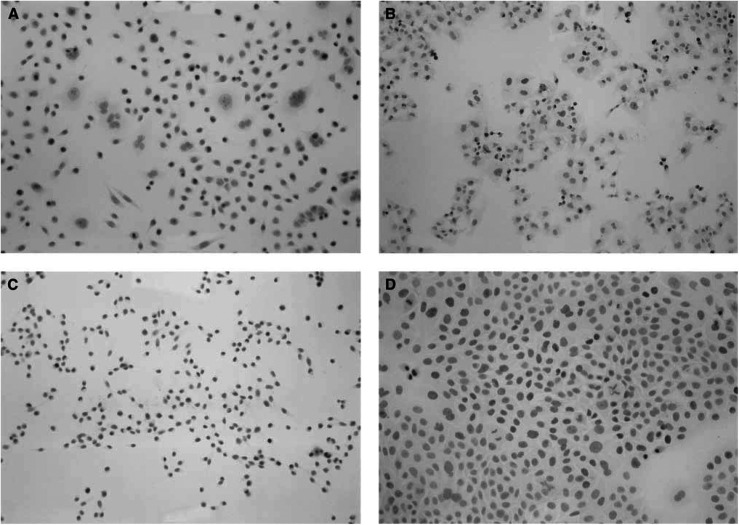
Immunohistochemical demonstration of the HMGA2 protein expression in pancreatic cancer cell lines. (**A**) AsPC-1 (Mayer's haematoxylin; original magnification × 200). (**B**) PANC-I (Mayer's haematoxylin; original magnification × 200). (**C**) MIA PaCa-2 (Mayer's haematoxylin; original magnification × 100). (**D**) BxPC-3 (Mayer's haematoxylin; original magnification × 200). Intense multifocal or diffuse HMGA2 nuclear immunoreactivity (brown colour) was characteristically observed in cancer cells.

**Figure 3:**
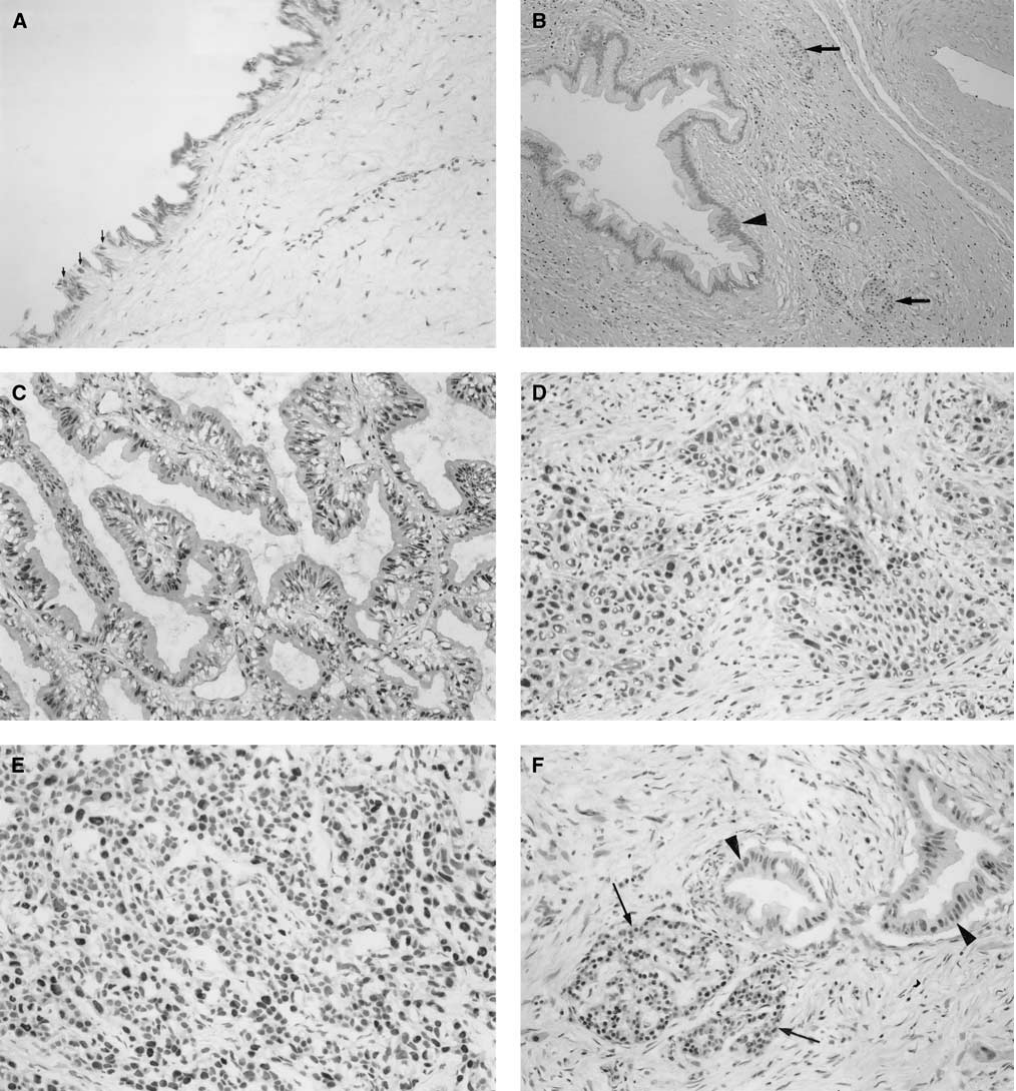
Immunohistochemical demonstration of the HMGA2 protein expression in surgically resected specimens of non-neoplastic pancreatic tissues and pancreatic carcinomas. (**A**) Non-neoplastic epithelial cells of the main pancreatic duct. A small proportion of duct epithelial cells show HMGA2 immunoreactivity (arrows). (Mayer's haematoxylin; original magnification × 200). (**B**) Epithelial cells of branch pancreatic duct and islets in chronic pancreatitis tissue. Islet cells showed intense and diffuse HMGA2 immunoreactivity (arrows), while epithelial cells of the branch pancreatic duct did not exhibit any detectable HMGA2 immunoreactivity (arrowhead) (Mayer's haematoxylin; original magnification × 100). (**C**) Primary pancreatic carcinoma exhibiting well-differentiated tubular adenocarcinoma (Mayer's haematoxylin; original magnification × 200). (**D**) Primary pancreatic carcinoma exhibiting adenosquamous carcinoma (Mayer's haematoxylin; original magnification × 200). (**E**) Metastatic lesion in the liver (Mayer's haematoxylin; original magnification × 200). Intense and multifocal or diffuse HMGA2 immunoreactivity was noted in all the pancreatic carcinomas (**C–E**). (**F**) Section including both carcinoma cells and islet cells (Mayer's haematoxylin; original magnification × 200). Islet cells showed intense and diffuse HMGA2 immunoreactivity (arrows), which was almost equivalent to that observed in carcinoma cells (arrowheads).

To determine whether the altered HMGA2 mRNA expression observed in pancreatic carcinoma is associated with alterations in protein expression, we analysed the expression of the HMGA2 protein by immunohistochemistry. Its expression was first analysed in four pancreatic cancer cell lines. Intense multifocal or diffuse HMGA2 nuclear immunoreactivity was characteristically observed in these cell lines (Figures 2A–D

). In both normal pancreas and chronic pancreatitis tissues, acinar cells did not exhibit any detectable HMGA2 immunoreactivity; however, a small proportion of duct epithelial cells showed faint HMGA2 immunoreactivity (Figure 3A

). On the other hand, in the endocrine region, islet cells showed intense and diffuse HMGA2 immunoreactivity (Figures 3B and F). In these cells, although HMGA2 immunoreactivity was localised mainly in the nuclei, faint staining was also observed within the cytoplasm. These results clearly indicated that the presence of the HMGA2 gene in non-neoplastic pancreatic tissue observed in the RT–PCR analysis reflects its expression in islet cells, together with its focal expression in duct epithelial cells. No significant difference in immunohistochemical findings was found between normal tissues and chronic pancreatitis tissues. When the expression of the HMGA2 protein in surgical specimens of carcinomas was then analysed, multifocally or diffusely distributed intense HMGA2 immunoreactivity was noted in all the pancreatic carcinoma specimens examined (Figures 3C–F). Intense nuclear staining was characteristically observed in the carcinoma cells. High-mobility group A2-positive carcinoma cells were observed regardless of the degree of differentiation (well/moderately or poorly differentiated tubular adenocarcinoma), histology type (tubular adenocarcinoma or adenosquamous carcinoma), or tumour site (primary or metastatic site). A strong correlation between HMGA2 overexpression and the diagnosis of carcinoma was noted (Fisher's exact probability, *P*<0.0001, Table 1).

## DISCUSSION

To evaluate the association between HMGA2 expression and the pathological diagnosis of pancreatic carcinoma, we investigated the expression of HMGA2 gene/protein in duct cell carcinoma and non-neoplastic tissue of the pancreas. High-mobility group A2 expression has been shown to be undetectable or to remain at low levels in normal adult tissues (Manfioletti *et al*, 1991; Zhou *et al*, 1995; Rogalla *et al*, 1996; Rommel *et al*, 1997; Hirning-Folz *et al*, 1998; Gattas *et al*, 1999). In the present study, however, a highly sensitive RT–PCR analysis revealed the expression of the HMGA2 gene in non-neoplastic pancreatic tissue, although its expression level was significantly lower than that in carcinoma. Immunohistochemical analysis indicated that the presence of the HMGA2 gene in non-neoplastic pancreatic tissue observed in the RT–PCR analysis reflects its abundant expression in islet cells together with its focal expression in duct epithelial cells. Thus, this study showed that the HMGA2 gene or protein is present even in normal pancreatic tissue. In HMGA2 immunohistochemical analysis, while only a small proportion of duct epithelial cells in the non-neoplastic tissue specimens showed HMGA2 immunoreactivity, a significantly higher proportion of carcinoma cells showed intense staining. In fact, a strong correlation between HMGA2 overexpression and the diagnosis of carcinoma was statistically verified. These findings indicate that an increased expression level of the HMGA2 protein is closely associated with the malignant phenotype in the pancreatic exocrine system, and accordingly, HMGA2 could serve as a potential diagnostic molecular marker for distinguishing pancreatic malignant cells from non-neoplastic pancreatic exocrine cells. A possible application of the results of the present study would be the determination of the HMGA2 gene and/or protein expression level in pancreatic juice collected at the time of endoscopic retrograde pancreatography. Using a sensitive and quantitative method such as competitive RT–PCR or immunoassay, the detection of even a small number of cancer cells could well be expected.

In order to evaluate the biological significance of the present results, it would be essential to understand the mechanisms by which the HMGA2 gene is involved in tumorigenesis, which unfortunately remain largely unclear. A clue to this issue was, however, provided by a recent report that transgenic mice carrying the HMGA2 gene developed pituitary adenomas (Fedele *et al*, 2002). These findings indicate that the high HMGA2 expression level has a critical role in neoplastic transformation of cells. Another clue was also demonstrated when antisense HMGA2 RNA was shown to prevent retrovirally induced neoplastic transformation of rat thyroid cells *in vitro* (Berlingieri *et al*, 1995). The interaction between HMGA2 and the AP-1 transcriptional complex is considered to be responsible for the activation of genes whose expressions are associated with carcinogenesis (Vallone *et al*, 1997), since thyroid neoplastic transformation is associated with a drastic increase in AP-1 activity. This AP-1 activity is blocked by suppressing HMGA protein synthesis *in vitro* (Battista *et al*, 1998). The absence or decreased AP-1 transcriptional activity, which is directly or indirectly regulated by HMGA proteins, would inhibit the expression of AP-1-dependent genes, such as those of vascular endothelial growth factor (VEGF), collagenase I (matrix metalloproteinase-1; MMP-1), and stromelisin (MMP-3), which are essential for neoplastic transformation of cells (Vallone *et al*, 1997). In fact, significant downregulations of these mRNA expression levels were demonstrated in the retrovirally infected thyroid cell lines expressing the antisense HMGA2 (Vallone *et al*, 1997). Considering that the overexpression of the AP-1 (Tessari *et al*, 1999; Meggiato *et al*, 2003), VEGF (Seo *et al*, 2000), MMP-1 (Ito *et al*, 1999), and MMP-3 (Bramhall *et al*, 1996) has been demonstrated in human pancreatic cancer, together with our results, the interactions among these molecules may play an important role in pancreatic neoplastic transformation *in vivo*, Further studies, including the determination of expression levels of these molecules in tissue samples, have yet to be carried out to further clarify this issue. Conversely, the HMGA2 gene has recently been shown not to be necessary for the malignant transformation of thyroid cells *in vivo* (Scala *et al*, 2001). This was demonstrated by comparing the frequency of radiation or papilloma virus E7 gene-induced thyroid carcinomas in mice carrying disrupted HMGA2 (pygmy mice) and that in mice carrying wild-type HMGA2 (Scala *et al*, 2001). Pygmy mice developed thyroid carcinomas with the same frequency as wild-type mice and furthermore, these two carcinomas generated in different mice showed no significant macroscopic and microscopic differences, indicating that HMGA2 is not sufficient for *in vivo* malignant transformation of thyroid cells (Scala *et al*, 2001). Several hypotheses could be considered to explain the discrepancy with the previous *in vitro* data, showing that HMGA2 is required for *v-mos*- and *v-ras-Ki*-induced cell transformations. One possible explanation would be that HMGA1, rather than HMGA2, may be required for thyroid cell transformation. This hypothesis is supported by the evidence that adenovirus carrying the HMGA1 gene in an antisense orientation induces programmed cell death in carcinoma cell lines derived from human thyroid, lung, colon, and breast cancers (Scala *et al*, 2000). We previously demonstrated that human pancreatic carcinoma expresses high HMGA1 levels (Abe *et al*, 2000, 2002), indicating that both HMGA2 and HMGA1 are overexpressed in this lesion. The expression of only one of the HMGA genes may be sufficient to lead epithelial cells of the pancreatic duct to exhibit the malignant phenotype. Further studies, such as the generation of HMGA1-knockout mice and subsequent analysis of their susceptibility to developing malignancies, need to be carried out in order to clarify the role of a single HMGA gene in carcinogenesis in a wide variety of epithelial tissues.

In conclusion, this study has clearly demonstrated that an increased expression level of the HMGA2 gene/protein is closely associated with the malignant phenotype in pancreatic exocrine tissue, suggesting that HMGA2 could play a vital role in tumorigenesis in the pancreatic exocrine system. The strong correlation between HMGA2 overexpression and the histological diagnosis of carcinoma indicates that the determination of the expression level of HMGA2 can be of great value in the diagnosis of pancreatic neoplasms.
